# Innovative Hydrocortisone Acetate and Pramoxine Hydrochloride Topical Cream Formulations for Anorectal Conditions: Enhanced In Vitro Release Profile

**DOI:** 10.3390/pharmaceutics17030348

**Published:** 2025-03-08

**Authors:** Onur Pinarbasli, Nurdan Atilgan, Ezgi Turkes, Nagehan Sarracoglu, Asuman Aybey Doganay

**Affiliations:** Research and Development Center, Ilko Pharmaceuticals, Ankara 06800, Turkey; natilgan@ilko.com.tr (N.A.); eturkes@ilko.com.tr (E.T.); nkeskin@ilko.com.tr (N.S.); aaybey@ilko.com.tr (A.A.D.)

**Keywords:** hydrocortisone acetate, pramoxine hydrochloride, anorectal disorders, in vitro release, HPLC

## Abstract

This study focuses on analyzing the in vitro release characteristics, as well as improving the penetration rate and stability of hydrocortisone acetate and pramoxine. This medication combination (hydrocortisone and pramoxine) is the first generic drug product utilized to alleviate minor pain, itching, swelling, and discomfort associated with anorectal conditions such as hemorrhoids. **Background/Objectives**: The developed novel formulations contain hydrocortisone acetate and pramoxine HCl as active ingredients, at least one solvent, at least one penetrating agent, at least one emulsifying agent, at least one surfactant, and at least one antimicrobial preservative, and pH values between 3.0 and 5.0, preferably between 3.5 and 4.5. **Methods**: Typical semi-solid dosage form quality control tests included appearance, identification, content homogeneity, pH, viscosity, assay, compounds of interest, microbiological testing, and in vitro release testing. In in vitro release testing, a series of formulations containing hydrocortisone acetate and pramoxine were tested for in vitro release across the Strat-M membrane using Franz diffusion cells methodology in comparison to a reference product (Pramosone Cream 2.5%). **Results**: Quantitative content of the release tests of the active ingredients in the cream, assay tests, antimicrobial preservative efficacy, and stability tests were carried out by high-sensitivity liquid chromatography. **Conclusions:** In conclusion, the cream formulations developed in this study have the potential to offer more effective treatment compared to reference products in terms of both in vitro release rates, and their reliability and validity were confirmed through validation studies.

## 1. Introduction

**Figure 1:**
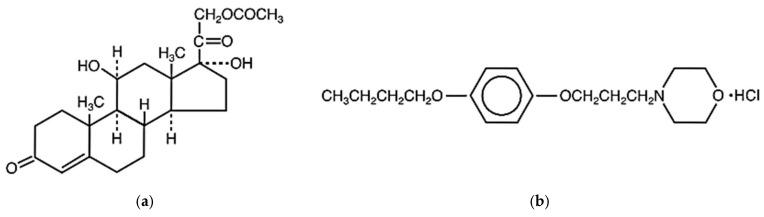
Structural formulas of (**a**) hydrocortisone acetate and (**b**) pramoxine HCl.

The corticosteroid hydrocortisone acetate is a synthetic preparation of the steroid hormone cortisol as 11β,17-Dihydroxy-3,20-dioxopregn-4-en-21-yl acetate (Figure 1) [1,2] and has vasoconstrictive, anti-inflammatory, and anti-pruritic effects. Hydrocortisone acetate administered topically has been reported to be beneficial in treating a variety of dermatological disorders, such as infantile eczema and atopic dermatitis, among a few other conditions. Furthermore, it seems that this material helps most, if not all, cases of inflammatory skin conditions [3,4].

Pramoxine is a topically acting local anesthetic as 4-[3-(*p*-Butoxyphenoxy)propyl]morpholine hydrochloride (Figure 1) [2,5]. Most local anesthetics have a lipophilic aromatic group and a hydrophilic amine group, linked by an intermediate chain, usually an ester or amide. Pramoxine, however, has a morpholine moiety acting as the linkage ether, making it structurally unique, with good potency and considerably fewer side effects. Pramoxine is a potent, rapidly acting surface anesthetic and antipruritic agent having a good safety profile. Because pramoxine and other topical anesthetics act on the same peripheral neural pathway (slow C fibers) that pain, itch, and thermal sensation share, they have both anesthetic and antipruritic effects [6,7,8].

This combination of medication [9] (hydrocortisone–pramoxine) is used to treat minor pain, itching, swelling, and discomfort caused by anorectal disorders like hemorrhoids and other problems of the anal area (such as anal fissures and itching) [10,11]. Both primary care doctors and gastroenterologists frequently see patients with anorectal problems. These illnesses are diverse and range from less serious conditions like hemorrhoids to more serious conditions like cancer. For this reason, it is critical that the doctor understands these disorders and is capable of performing a proper history and physical examination. Hemorrhoids, anal fissures, fecal incontinence, proctalgia fugax, excessive perineal descent, and pruritus are the most prevalent anorectal disorders [12,13].

In vitro release testing (IVRT) is a useful test to assess product similarity under certain scale and post approval changes for semisolid products. The FDA Guidance on Scale up and Post Approval Changes for Semisolid (SUPAC-SS) describes suitable conditions for this testing.

Semi-solid pharmaceutical product testing, such as that developed in this study, is generally divided into two categories: (1) those that assess general quality characteristics, i.e., product quality tests, and (2) those that assess product performance, e.g., using an IVRT/IVPT (in vitro permeation test) method. Product quality tests describe the formulation’s physicochemical and/or structural characteristics (such as pH and particle size/morphology). Product performance tests, on the other hand, evaluate how a medication product performs under particular circumstances, which may offer insights into how well it works in vivo, characterize, and/or compare the linear (steady-state) drug release rate from semisolid dosage forms. By comparing the steady-state drug release rate of an approved (reference standard) and a potential generic (test) product, the validated IVRT method characterizes the impact of manufacturing differences, processes, and/or formulations on a drug product. In some cases, this can support a demonstration of bioequivalence, and in other cases, it can support a demonstration of the product’s safety and/or efficacy [14].

The analytical techniques, such as high-performance liquid chromatography (HPLC), must be precise, accurate, and specific for the drug substance in the receptor solution during administration. When the literature is examined, there are quantitative test methods for hydrocortisone acetate [15,16,17,18,19] and promaksin HCl separately, most of which are HPLC techniques [20,21,22,23].

The number of studies including the HPLC method in which the antimicrobial preservative can also be distinguished in the cream formulation containing both agents is in the minority [24]. The use of established analytical techniques with multi-point calibration curves is recommended. Validation of analytical technique for IVRT sample analysis should be performed in accordance with ICH guidelines [25,26,27,28].

This study addresses the need to effectively treat topical application conditions, which are local in nature, systemically with the same treatment regimen. In this way, a cream formulation was developed containing hydrocortisone acetate and pramoxine with improved in vitro release compared to the commercially available reference product Pramosone Cream 2.5% of Ferndale Laboratories Inc., USA [13].

The aim of this study was to prevent an anorectal disorder by achieving a better therapeutic effect than commercially available compositions containing the same active ingredients in the same ratio and excipients in different ratios, increase skin penetration to reach therapeutic levels, exhibit improved stability upon in vitro release and long-term storage, and not significantly degrade during storage. Thus, the newly developed stable formulation that enhances both the extent and speed of in vitro release of hydrocortisone acetate and pramoxine through the skin is expected to be more effective for treating localized conditions and, even more significantly, will substantially improve the likelihood of achieving systemic treatment through topical application.

## 2. Materials and Methods

### 2.1. Materials

For the formulation study, hydrocortisone acetate was obtained from Tianjin Tianyao Pharmaceuticals; pramoxine HCl was obtained from Syn-Tech Chem & Pharmaceuticals; isopropyl palmitate (Nikko Chemicals, Tokyo, Japan); triethanolamine lauryl sulfate, stearic acid (Merck, Darmstadt, Germany), polyoxyl 40 stearate, lanolin alcohol, liquid paraffin (Rchemie, Turkey), and cetyl alcohol (Sigma, St. Gallen, Switzerland); potassium sorbate (Celanese, Sulzbach, Germany); sorbic acid (Merck, Germany); and propylene glycol (BASF, Nienburg, Germany) were used as inactive ingredients in the formulation. All these inactive excipients used in the formulation were pharmaceutical grade. Distilled water was obtained from Agilent.

For analytical validation and the IVRT study, hydrocortisone acetate (USP Reference Standard), pramoxine HCl (USP Reference Standard), potassium sorbate, and sorbic acid, were used throughout the analysis. All solvents were HPLC-grade including acetonitrile (J.T. Baker, Shanghai, China), ethanol (J.T. Baker), ortho-phosphoric acid (Merck, Germany), and potassium dihydrogen phosphate (Merck, Germany). High-purity water was prepared using an ELGA Purelab Classic purification system.

Instrumentation for the HPLC system was used from Waters (Waters, Milford, MA, USA), including the Waters E2695 Separations Module with a Waters 2489 UV–Visible detector. The chromatographic data were collected and processed using Empower 2 software FR5.

Chromatographic conditions for the HPLC separations were conducted using an ODS 3V GL Sciences (250 × 4.6 mm, 5 µm) column at 30 °C. Mobile phase A is a phosphate buffer solution with a pH value of 7.5 and mobile phase B is acetonitrile. The flow rate is 1.8 mL min^−1^. The injection volume is 50 μL, and the analytes are detected at 224 nm. The total analysis run time is 10 min.

IVRT Franz diffusion cell system conditions of the in vitro release tests were conducted using vertical Franz diffusion cells methodology. Six individual cells, standard open cap ground glass surface with 15 mm diameter orifices, 35 mL volume capacity, and total diameter of 25 mm. The phosphate buffer solution had a pH value of 7.4 and ethanol. Membrane with properties closest to human skin Strat-M membrane filter (Merck Millipore).

### 2.2. Methods: Analytical Method and Validation Studies

In this study, method validation was performed for hydrocortisone acetate–pramoxine HCl cream by HPLC method for stability, finished product quantification, in vitro release, and antimicrobial preservative testing. The colon is Inertsil ODS 3V (250 × 4.6) mm, 5 µm; the flow rate is gradient and 1.8 mL/min; the wavelength is 224 nm; the injection volume is 50 microliters; the colon temperature is 30 °C; the sample temperature is 20 °C; the solvent is water–acetonitrile (40:60); for the mobile phase tampon, 8.71 g of dipotassium hydrogen phosphate is dissolved with 1000 mL of water, pH adjusted to 7.5 ± 0.05 with orthophosphoric acid, filtered through a 0.45 µm PTFE (Sartorius, Gottingen, Germany) filter, and degassed for 10 min.

### 2.3. Preparation of Standard Stock

Preservative Stock Solution (Sorbic acid): 20 mg Sorbic acid working standard is weighed into a 20 mL balloon jug. After adding approximately 10 mL of solvent, it is kept in an ultrasonic bath for 20 min at 15 °C (AL 04-12, Advantage Lab, Belgium, Switzerland) and the volume is completed with solvent.

### 2.4. Preparation of Standard

Weigh 30 mg of hydrocortisone acetate and 12 mg of pramoxine HCl working standard into a 200 mL balloon jug. After adding approximately 60 mL of solvent, add 2 mL of the preservative solution prepared above. After 20 min in the ultrasonic bath, the volume is topped up with solvent. The solution is filtered through a 0.45 µm PVDF filter, vitalized, and transferred to HPLC (C HID = 0.15 mg/mL, C PRA = 0.06 mg/mL, C KOR = 0.01 mg/mL).

### 2.5. Preparation of Sample Solution for Validation

Approximately 600 mg of cream sample is weighed into a 100 mL beaker. Approximately 60 mL of solvent is added and stirred in a magnetic stirrer at 600 rpm for 2 h. Then the solution in the beaker is transferred to a 100 mL balloon jug, and the volume is topped up with solvent. The final solution was filtered through a 0.45 µm PVDF filter and transferred to an HPLC vial.

### 2.6. Analytical Method Validation

The suggested approach was validated following the International Conference on Harmonization’s (ICH) recommendations for validating analytical methods. The method of analysis was validated using the following parameters: specificity, linearity, accuracy, precision robustness, and solution stability.

### 2.7. Specificity

A specificity test was conducted to evaluate the method’s ability to measure the active ingredient of interest in the sample. The specificity test is performed to determine the ability of the method to measure only the substances intended to be measured in the analyzed sample. To measure the specificity of the method, the selectivity and stress studies listed in Table 1 are performed.

### 2.8. Linearity

Linearity is crucial to demonstrate that sample solutions fall within a concentration range where the analyte response is directly proportional to concentration. To prove the linear response relationship, the peak areas of solutions prepared at six different concentrations (20%, 40%, 60%, 80%, 100%, and 120%) are measured. The correlation coefficient between concentrations and areas should not be lower than 0.99. Residuals of each component should be within ±10% with respect to the 100% concentration response.

### 2.9. Precision

System precision refers to the degree of consistency or reproducibility of the analytical method when applied to a sample over a series of measurements. Repeatability and intermediate precision were used to evaluate the method precision. Intra-day precision (repeatability) and inter-day precision (intermediate precision) were assessed by testing six sample solutions. The inter-day precision samples were prepared by different analysts using different HPLC systems on different days. Table 2 summarizes the definitions, procedures, and acceptance criteria for system precision, repeatability, and intermediate precision.

### 2.10. Accuracy

Accuracy refers to the degree to which the results of an analytical method agree with the true or known value of the analyte being measured. Raw material solutions containing the same amount of placebo were prepared at 80%, 100%, and 120% levels. A total of nine samples were prepared, with three for each level. The acceptance criteria of the accuracy parameter must fulfill the following conditions.
**Acceptance Criteria**Agreement between Standard 1 and Standard 2 should be within 98.0–102.0%. RSD between areas from six injections of Standard 2 should not exceed 2.0%. % recovery of each studied sample should be between 95.0–105.0%. RSD between the % recovery values for the active substance should not be greater than 2.0%. The symmetry factor of the main peaks should not exceed 2.0.

### 2.11. Robustness

Robustness in analytical validation refers to the ability of an analytical method to remain unaffected by small, deliberate variations like flow rate, wavelength and column temperature in method parameters and conditions. The acceptance criteria of the robustness parameter must fulfil the following conditions.
**Acceptance Criteria**Agreement between Standard 1 and Standard 2 should be within 98.0–102.0%. Relative standard deviation (%RSD) between the areas obtained from six injections of Standard 2 should not exceed 2.0%. The results of each sample studied must be within the specification limits. The results obtained must be compared with the repeatability results. The symmetry factor of the main peaks should not be greater than 2.0.

### 2.12. Solution Stability

Solution stability in analytical validation refers to the ability of a solution containing an analyte (or analytes) to maintain its chemical integrity and concentration over time under specified storage conditions (at room temperature 25 °C). Acceptance criteria is a solution is considered stable if the agreement between the solutions is 98.0–102.0%.

### 2.13. Drug Formulation Studies

#### Preparation of Cream Formulation

**Figure 2:**
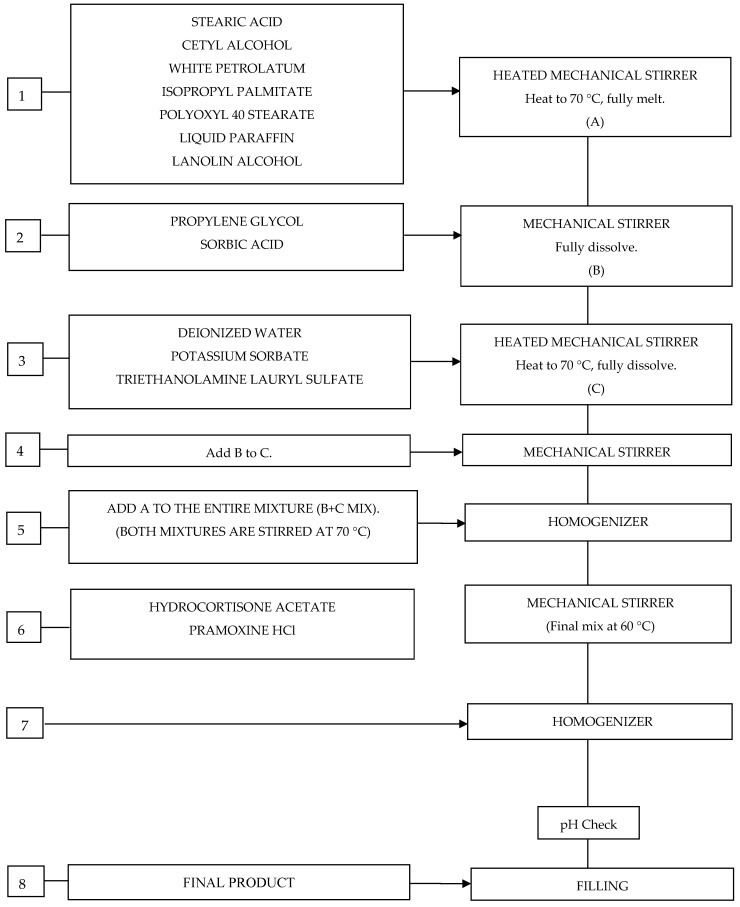
Production process flowchart.

In pharmaceutical technology, the production of creams and ointments is based on the separate preparation and mixing of two phases, the water phase and the oil phase. An oil/water emulsion is prepared by mixing the phases. Since a smooth cream was desired to be obtained, the prepared cream was made smooth with a homogenizer. Finally, the pH was measured. The details of the production of the cream formulation, the contents of which are given in Table 3, are given in the production process flow chart below (Figure 2). The critical parameters of the production process were determined as the addition of active substances and excipients to the optimal phase, mixing speed and duration, temperature, and homogenization speed.

### 2.14. In Vitro Release Testing

**Figure 3:**
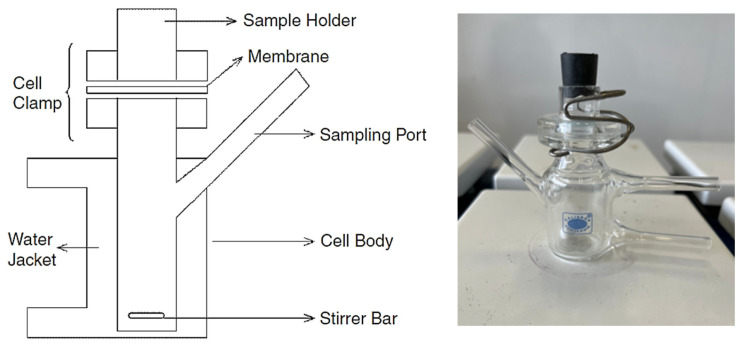
Schematic representations of Franz dissolution apparatus.

The in vitro release rate is routinely performed using the Franz diffusion cell methodology. IVRT is a useful test for semi-solid products to assess product similarity between different formulation studies, according to reference products. Using Franz diffusion cells, several formulations, including hydrocortisone acetate and pramoxine, were evaluated for in vitro release via the Strat-M membrane, an artificial membrane closest to human skin, in contrast to the reference product (Pramosone Cream %2.5). This cell, which was first described by Franz’s dissolving apparatus in 1978, has a cylindrical receptor chamber that may be mixed with a magnetic stir bar and a small donor compartment, as seen in Figure 3.

The Franz diffusion cell system is the equipment utilized in IVRT. A donor compartment and a receiver compartment make up a Franz diffusion cell. It is made up of six separate cells. Every cell features a conventional open-cap ground glass surface with orifices measuring 15 mm in diameter, a volume capacity of 35 mL, and a total diameter of 25 mm. There is a clamp holding the top and bottom sections together. Receptor fluid (pH 7.4 and 0.1 M potassium phosphate buffer–ethanol) in a 70:30 ratio fills the area of the cell beneath the installed membrane entirely, ensuring that the fluid is in contact with the membrane. Using a magnetic stirrer, the receptor fluid is swirled. The semisolid formulation, weighing about 400 mg, is evenly spread out across a synthetic membrane and left open to stop compositional changes and solvent evaporation. To produce a sufficient release profile and calculate the drug release rate, multiple sample times (at least 5) over a suitable time span are advised. Following the removal of each aliquot, analysis is usually performed using the validated technique HPLC. The cell was replenished with fresh media in an amount equal to the removed aliquot volume after each aliquot was removed. The total amount of medication released at 30, 60, 120, 180, 240, and 300 min is recorded, with the results stated in mg/cm^2^.

Using HPLC analysis, the amounts of pramoxine and hydrocortisone acetate in the samples were determined. HPLC was specifically performed using an ODS 3V column (250 × 4.6 mm, 5 µm) and a mobile phase of phosphoric acid buffer–acetonitrile (40:60) at pH 7.5. Using established calculations based on the total transference of pramoxine and hydrocortisone acetate across the skin after five hours, flux rates were determined.

Thus, flux rates, *F*, were computed according to the following formula:

$$F=\frac{D\times V}{t\times A}$$

where *D* is the concentration of the active substances in the receptor well after incubation time *t*, *V* is the volume of the receptor well, and *A* is the surface area of membrane.

The conditions used for IVRT for the example compositions of the invention are as follows:
**Receptor medium**pH 7.4 buffer–ethanol (70:30)**Speed**500 rpm **Membrane**Strat-M membrane filter (Merck Millipore)**Sample application**~400 mg **Temperature**32 ± 0.5 °C**Sampling times**0.5, 1, 2, 3, 4, and 5 h (Six time points)
pH 7.4 Buffer Preparation: 13.6 g of potassium dihydrogen phosphate is dissolved in 1 L of water; the pH value is adjusted to 7.4 with 10 N of NaOH.

## 3. Results

### 3.1. Analytical Methods and Validation Studies

#### 3.1.1. Specificity

A specificity test was performed to determine the ability of the method of measuring only the substances that were aimed to measure in the sample analyzed.

#### 3.1.2. Selectivity

**Figure 4:**
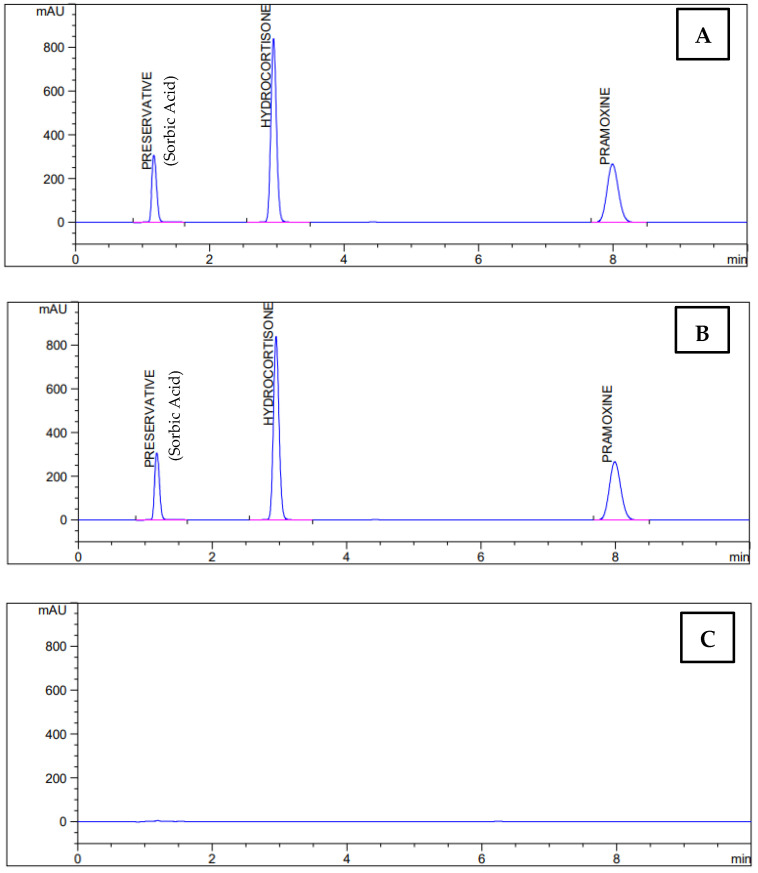
Selectivity chromatograms: (**A**) sample, (**B**) standards, and (**C**) placebo.

For selectivity testing, standard, sample, mobile phase, placebo, antimicrobial preservative (potassium sorbate and sorbic acid) were injected (Figure 4). Spectra of the injected solutions were taken.
**Substance****Observations****Hydrocortisone Acetate**Standard and sample spectra are similar.**Pramoxine HCl**No peaks at the retention time of active compounds in the standard and sample**Antimicrobial Preservative Peaks**


#### 3.1.3. Stress Studies

For stress test, samples exposed to stress conditions (under the conditions written in the Section 2) were injected in distinct durations (Table 4).
**Observation****Description****Non-overlapping Peaks**No overlapping occurred between the exterior peaks formed for hydrocortisone acetate and pramoxine HCl during stress studies, indicating successful distinction.**Effect of Stress Conditions**Hydrocortisone acetate, pramoxine HCl, and the antimicrobial preservative were affected by a basic medium and 80 °C temperature, leading to deformation due to water evaporation. The antimicrobial preservative was also sensitive to oxidative conditions and humidity.**Method’s Sensitivity to Stress Conditions**The method effectively detected transitions in the sample under various stress conditions, demonstrating its stability-indicating properties.

#### 3.1.4. Linearity

**Figure 5:**
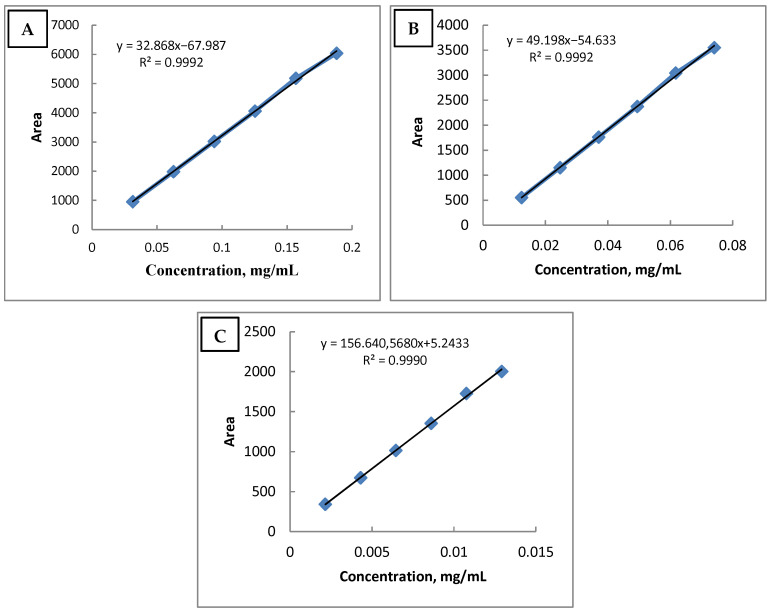
Hydrocortisone acetate (**A**), pramoxine HCl, (**B**) and antimicrobial preservative substance (sorbic acid) (**C**) linearity graph.

As shown in the Table 5 and graphics below (Figure 5), the correlation coefficient between concentrations and areas is not lower than 0.99, proving the linearity of the method.

### 3.2. Precision

#### 3.2.1. System Precision

A total of six replicate Injections of standard solution were performed. Areas and relative standard deviation value between them and system suitability was measured. System precision was demonstrated (Table 6).

#### 3.2.2. Repeatability

The parameters of repeatability (intra-day precision) were determined on six samples, and all results are summarized in Table 7 and high repeatability is demonstrated across all substances.

#### 3.2.3. Intermediate Precision

The parameters of reproducibility (inter-day precision) were determined on six samples and all results are summarized at Table 8 and high inter-day precision is demonstrated across all substances.

### 3.3. Accuracy

Accuracy of Method: Raw material solutions were prepared by adding active drug substance to placebo at 20%, 100%, and 120% levels. A total of nine samples were prepared, three for each level, and the results of each sample are in the specification limits (Table 9). The accuracy of the method was demonstrated by this study.

#### Accuracy Accepted Criteria Summary


**Parameter**

**Value**

**Percent Agreement Between Standards**


** - Hydrocortisone Acetate**
101.0%
** - Pramoxine HCl**
101.9%
** - Preservative Substance**
101.7%
**Relative Standard Deviation (%RSD) from Standard-2 Injection**


** - Hydrocortisone Acetate**
1.10%
** - Pramoxine HCl**
0.89%
** - Preservative Substance**
0.68%
**Symmetry Factors**
Not more than 2.0
**Specification Limits**


** - Sample Results (95.0–105.0%)**
Within limits
** - RSD Values Between Percent Recoveries (2.0%)**
Within limits

### 3.4. Robustness

Robustness of the method against variations was tested by assay analyses by changing analysis conditions (flow rate, column temperature, and wavelength). Each of the changing condition results compared with normal conditions was found to be favorable (Table 10).

#### Comparison of Robustness and Repeatability Test


**Acceptance Criteria**
Percent agreement between Standard 1 and Standard 2 is within 98.0–102.0%.Relative standard deviation (RSD%) of areas obtained from six replicate injections of Standard 2 is less than 2.0%.The result of each sample studied was examined within the specification limits.The assay method shows resistance to changes in column temperature, flow rate, and wavelength.

### 3.5. Solution Stability

According to the stability of the solutions analysis results, it is demonstrated that the standard solution and sample solution of hydrocortisone acetate and pramoxine HCl are stable at room temperature (25 °C) for 18 h according to acceptance criteria 95.0–105.0% (Table 11).

Hydrocortisone acetate–pramoxine HCl 2.5–1% cream assay and preservative substance method validation was completed successfully by carrying out specificity, linearity, precision, repeatability, accuracy, and robustness parameters. All the results match the limits. It can be used in in vitro release, quantitative tests, and stability tests.

### 3.6. Drug Formulation Studies Evolution

The standard quality control tests for semisolid dosage forms used in pharmaceutical products include appearance, uniformity of content, pH, viscosity, assay, related compounds, and in vitro release testing. A straightforward, dependable, and repeatable in vitro release rate technique can direct formulation development, support batch-to-batch quality and stability monitoring, and regulate the cosmeceutical manufacturing process—all in a manner akin to the dissolution testing of oral dosage forms. It is especially helpful in determining how modifications to the drug’s composition, excipients, and manufacturing method have an impact on the final product.

In order to improve the in vitro release profile of the semi-solid product, the formulation development studies focused on the penetration agent, emulsifying agent and pH values of the finished product, and trial studies were carried out on these parameters and tried to be optimized (Table 12).

The formulation composition of all trials should contain the active substances hydrocortisone and pramoxine; at least one penetrating agent; at least one surfactant; at least one emollient/emulsifying agent; at least one antimicrobial preservative; and at least one solvent, as shown in Table 12. Wherein the pH of the finished product is between 3 and 5, preferably between 3.5 and 4.5.

In the first and second formulation trials, the ratios of emulsifying agent stearic acid and white petrolatum were changed, and the penetration agent and surfactant ratios were kept constant. The pH of the finished product was measured, and the first formulation was 2.5, and the second formulation was 5.2. In both trials, it was observed that the products underwent pH-dependent phase separation during storage in room conditions. Depending on the phase separation, as shown in Table 13, out-of-limit findings due to variation were detected for all active ingredients in the finished product content, uniformity and assay analysis results. It was not deemed appropriate to initiate an in vitro release study of these unstable trials.

In the third formulation trial, in addition to the emulsifying agent stearic acid and white vaseline, the penetrating agent was increased from 2% to 5%, and lanoline alcohol, another emulsifying agent, was removed from the formulation; then the pH measured was within the recommended range, i.e., 4.0, and no phase separation was observed. However, in this study, although all content tests were appropriate (Table 13), the viscosity value was quite high compared to the reference product (650 e3cp) and not deemed appropriate to initiate an in vitro release study.

In the fourth and fifth formulation trials, the emulsifying agent stearic acid was kept constant at 9%, and white vaseline 12%, as in the 3rd formulation. The rate of penetrating agent isopropyl palmitate was reduced to 2%; lanoline alcohol was not used in FT-4, while it was used at 1% in FT-5. pH was measured within the recommended range, i.e., 4.0, and as can be seen in Table 13, the content test results for both formulations were found to be appropriate and in vitro release studies were initiated.

In the sixth formulation trial, emulsifying agent stearic acid and white petrolatum ratios were decreased, penetrating agent isopropyl palmitate ratio was increased by 4%, and the amount of linoline alcohol was kept constant at 1% based on the FT-4 and FT-5 formulas. pH was measured as 4.5 in the desired range and in vitro release studies were initiated since all content tests were found to be suitable.

Finally, in the seventh formulation study, in order to examine the effect of the penetration agent isopropyl palmitate and emulsifying agent lanoline alcohol, lanoline alcohol was removed from the formulation over FT-4 and FT-5, and the ratio of isopropyl palmitate was reduced to 1%. pH was measured in the desired range, i.e., 4.0, but in vitro release studies were not initiated because the viscosity (63 e3cp) value was obtained considerably lower than the reference product result, although the content tests were found to be appropriate.

**Figure 6:**
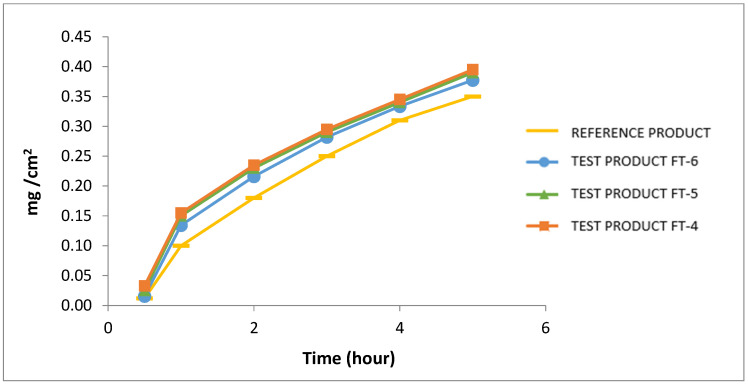
In vitro release profile for hydrocortisone acetate in comparison with reference product (Pramosone Cream 2.5%).

**Figure 7:**
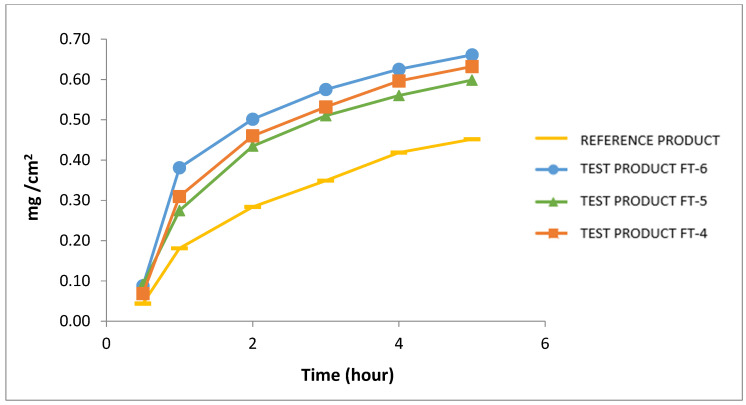
In vitro release profile for pramoxine HCl in comparison with reference product (Pramosone Cream 2.5%).

When these trials and formulation studies are evaluated, it is seen that the penetration agent, isopropyl palmitate, and emulsifying agent, linoline alcohol, have important roles in obtaining the optimum cream formulation depending on pH. The ratios of these critical excipients in the formulation are seen to offer distinctive features and provide superiority to the reference product as seen in Figure 6 and Figure 7 in the in vitro release test profiles carried out against the reference product.

When compared to a commercially available reference product, the innovative formulations first generic product was more successful at improving hydrocortisone acetate and pramoxine in vitro release through the skin, as indicated by in vitro release profiles.

Pramoxine’s fast and high in vitro release, combined with its analgesic properties, allows for quick pain relief for the patient. This was assessed as the developed product’s unexpected and technological consequence. Control test results of the formulation trial of hydrocortisone acetate and pramoxine HCl are given in Table 13.

## 4. Discussion

The present study successfully developed and validated novel topical cream formulations containing hydrocortisone acetate and pramoxine HCl for the treatment of anorectal conditions. By optimizing the formulation parameters, including penetration enhancers, emulsifiers, and pH levels, the study demonstrated an improved in vitro release profile compared to the commercially available reference product, FDA approved Pramosone Cream 2.5%.

A critical finding of this study is the enhanced transdermal penetration and release rate of the active ingredients. The optimized formulation, which incorporates isopropyl palmitate as a penetration enhancer and a carefully balanced emulsifier system, facilitated faster and more efficient drug diffusion across the Strat-M membrane. This was evident in the IVRT results, where the newly developed formulations exhibited superior release characteristics, leading to a potentially more rapid onset of therapeutic effects. This advantage is particularly significant for patients experiencing acute pain and discomfort associated with anorectal disorders, as pramoxine’s rapid action can provide swift symptomatic relief.

The analytical validation studies confirmed the reliability and reproducibility of the formulation’s quality attributes. Parameters such as specificity, precision, accuracy, robustness, and stability were assessed in accordance with ICH guidelines, ensuring that the methodology employed for evaluating the formulations is both scientifically sound and industry-compliant. Notably, the formulation maintained its stability over the study period, indicating its potential for long-term clinical application without significant degradation of active ingredients.

Another key observation from this study was the importance of formulation pH in maintaining product integrity. Initial formulations FT-1 and FT-2 with extreme pH values exhibited phase separation over time, compromising uniformity and release consistency. However, by adjusting the pH to an optimal range of 3.5 to 4.5, which is also ideal for antimicrobial protection, phase stability was achieved, resulting in robust formulations of FT-4, FT-5, and FT-6, which are suitable for therapeutic application. Depending on the emulsifying agent and penetrating agent contents used in FT-3 and FT-7 formulations, the viscosity values were not found appropriate. In the sixth formulation trial FT-6, emulsifying agent stearic acid and white petrolatum ratios were decreased, penetrating agent isopropyl palmitate ratio was increased by 4% and the linoline alcohol amount was kept constant at 1% based on the FT-4 and FT-5 formulas. pH was measured as 4.5 in the desired range and in vitro release studies were initiated since all content tests were found to be suitable.

When compared with the reference product, the innovative formulations FT-4, FT-5, and FT-6 demonstrated a higher and more consistent release rate of hydrocortisone acetate and pramoxine HCl. Based on the release profiles, FT-6 was identified as the most optimal formulation. This suggests that the optimized cream may offer improved bioavailability, leading to enhanced clinical efficacy. Moreover, the validated high-performance liquid chromatography (HPLC) method provided precise quantification of the active ingredients and antimicrobial preservatives, further supporting the formulation’s reliability.

In conclusion, this study presents a promising advancement in the formulation of hydrocortisone acetate and pramoxine HCl creams for anorectal conditions. The enhanced in vitro release profile, combined with formulation stability and validated analytical methodologies, positions this product as a strong candidate for improved patient outcomes. With this study, which we think will make a significant contribution to the literature, future studies may focus on in vivo evaluations to further substantiate these findings and establish bioequivalence with existing commercial formulations.

## Figures and Tables

**Table 1:** The selectivity and stress studies conditions.

Test Step	Conditions	Analysis	Acceptance Criteria
**Selectivity Testing**	Standard, sample, spiked sample, placebo, mobile phase, impurities injected	Chromatogram of the injected solutions are compared.	- No peaks from solvent, placebo, impurities, or mobile phase at the retention time of active compounds in the standard and sample chromatograms - Standard and sample chromatograms should be similar
**Stress Studies**	Acidic Medium: 1 N HCl Basic Medium: 1 N NaOH Oxidation: 1% H_2_O_2_ Temperature: 80 °C, 7 days Temperature and Humidity: 40 °C, 75% RH, 21 days	Samples exposed to below stress conditions were injected in distinct durations. Chromatograms of injected samples were recorded.	There should not be any overlap between exterior peaks formed during stress studies

**Table 2:** System precision, repeatability, and intermediate precision studies conditions.

Test	Definition	Procedure	Acceptance Criteria
**System** **Precision**	Measures the consistency or reproducibility of an analytical method when applied to the same sample under identical conditions.	Six consecutive injections are performed. Areas and %RSD are calculated; symmetry factor and theoretical plate number are reported.	- %RSD between areas from six consecutive injections should not exceed 2.0%. - Symmetry factor of the main peaks should not exceed 2.0. - Theoretical plate number should not be less than 2000
**Repeatability**	Evaluates the consistency of the method when analyzing the same sample multiple times under the same conditions.	Agreement between Standard 1 and 2 is assessed. %RSD for six injections is calculated, and each sample result is recorded.	- Agreement between Standard 1 and 2 should be between 98.0% and 102.0%. - %RSD for six injections should not exceed 2.0%. - Symmetry factor should not exceed 2.0.
**Intermediate Precision**	Assesses variability of results when the same method is applied under different conditions within the same laboratory (by different analysts using different HPLC systems).	%RSD from repeatability and precision tests is compared.	- Agreement between Standard 1 and 2 should be between 98.0% and 102.0%. - %RSD from six injections should not exceed 2.0%. - %RSD from 12 results should not exceed 2.0%.

**Table 3:** Composition of the product.

Ingredients	Function
Hydrocortisone acetate	Active substance
Pramoxine HCl	Active substance
Stearic acid	Emulsifying agent
Cetyl alcohol	Emollient/emulsifying agent
White vaseline	Oil phase carrier
Isopropyl palmitate	Penetration agent
Polyoxyl 40 stearate	Emulsifying agent
Propylene glycol	Solvent (cosolvent)
Potassium sorbate	Antimicrobial preservative
Sorbic acid	Antimicrobial preservative
Liquid paraffin	Emollient
Lanoline alcohol	Emulsifying agent
Trietanolamin lauryl sulfate	Surfactant
Deionized water	Aqueous phase carrier

**Table 4:** Stress study results.

Stress Condition	Hydrocortisone Acetate, %	Pramoxine HCl, %	Antimicrobial Preservative Substance, %
**1 N HCl—24th hour**	98.6	105.0	94.0
**1 N NaOH—24th hour**	0.14	40.2	59.2
**1% H_2_O_2_—24th hour**	104.6	102.8	88.4
**Temperature 40 °C; 75% RH—14th day**	108.6	107.8	87.1
**Temperature 80 °C—5th day**	244.4	238.6	159.4

**Table 5:** Linearity: hydrocortisone acetate, pramoxine HCl, and antimicrobial preservative substance.

Substance	Concentration (mg/mL)	Regression Equation	Correlation Coefficient (R^2^)
**Hydrocortisone Acetate**	0.031–0.188	Y = 32,868x − 67.987	0.9992
**Pramoxine HCl**	0.012–0.074	Y = 49,198x − 54.633	0.9992
**Antimicrobial Preservative Substance**	0.002–0.013	Y = 156,640.568x + 5.2433	0.9990

**Table 6:** System precision results: hydrocortisone acetate, pramoxine HCl, and preservative substance.

Substance	Average Symmetry Factor	Average Theoretical Plates	Average Retention Time (Minutes)	Standard Deviation (SD)	%Relative Standard Deviation (RSD%)	Confidence Interval (95%)
**Hydrocortisone Acetate**	1.08	6438	2.98	0.004	0.14	0.003
**Pramoxine HCl**	1.08	10,097.05	8.03	0.015	0.19	0.012
**Preservative Substance**	1.26	1347.60	1.17	0	0	0

**Table 7:** Hydrocortisone acetate–pramoxine HCl 2.5–1% Cream assay results.

Parameter	Hydrocortisone Acetate	Pramoxine HCl	Preservative Substance
**Percent Agreement Between Standard**	100.0%	100.1%	99.9%
**Average, %**	99.3%	106.6%	92.4%
**Standard Deviation (SD)**	1.45	1.73	1.24
**Relative Standard Deviation (%RSD)**	1.46	1.62	1.34
**Confidence Interval (95%)**	99.3 ± 1.16	106.6 ± 1.38	92.4 ± 0.99

**Table 8:** Hydrocortisone acetate–pramoxine HCl 2.5–1% cream assay results.

Parameter	Hydrocortisone Acetate	Pramoxine HCl	Preservative Substance
**Percent Agreement**	98.8%	100.3%	99.5%
**Average, %**	101.3%	107.6%	94.7%
**Standard Deviation (SD)**	1.58	0.51	1.20
**Relative Standard Deviation (%RSD)**	1.56	0.47	1.27
**Confidence Interval (95%)**	101.3 ± 1.27	104.7 ± 0.40	94.7 ± 0.96

**Table 9:** Accuracy: hydrocortisone acetate–pramoxine HCl 2.5–1% cream sample results.

Sample Name	Recovery of Hydrocortisone Acetate (%)	Recovery of Pramoxine HCl (%)	Recovery of Preservative Substance (%)
**% 20-1**	98.3	95.8	102.5
**% 20-2**	97.4	95.7	101.5
**% 20-3**	96.9	95.1	100.9
**% 100-1**	101.5	96.6	103.8
**% 100-2**	100.7	96.3	101.2
**% 100-3**	100.8	95.7	102.4
**% 120-1**	100.1	97.9	98.5
**% 120-2**	101.8	98.2	101.6
**% 120-3**	101.8	97.8	100.9
**Average**	**99.9**	**96.0**	**101.5**
**SD**	**1.91**	**1.90**	**1.45**
**%RSD**	**1.92**	**1.97**	**1.43**
**Confidence Interval (95%)**	**99.9 ± 1.25**	**96.0 ± 1.24**	**101.5 ± 0.95**

**Table 10:** Robustness and repeatability test results: hydrocortisone acetate, pramoxine HCl, and preservative substance.

Analysis Name	Retention Time (Minutes)	Assay Percentage	Symmetry Factor	Number of Theoretical Plates
**Hydrocortisone Acetate**				
Normal Conditions	2.98	99.3	1.08	6438
Flow Rate: 1.7 mL/min	3.14	101.2	1.06	6447
Flow Rate: 1.9 mL/min	2.81	101.0	1.06	6013
Column Temperature: 28 °C	2.98	100.8	1.06	6411
Column Temperature: 32 °C	2.94	100.7	1.09	6491
Wavelength 222 nm	2.97	100.4	1.08	6377
Wavelength 226 nm	2.06	99.8	1.07	6374
**Pramoxine HCl**				
Normal Conditions	8.03	106.6	1.08	10,097
Flow Rate: 1.7 mL/min	8.42	108.4	1.05	9608
Flow Rate: 1.9 mL/min	7.55	108.0	1.05	9151
Column Temperature: 28 °C	7.99	107.9	1.08	9951
Column Temperature: 32 °C	7.95	107.8	1.07	10,279
Wavelength 222 nm	7.95	105.9	1.09	9891
Wavelength 226 nm	7.92	105.4	1.08	991
**Preservative Substance**				
Normal Conditions	1.17	92.4	1.26	1348
Flow Rate: 1.7 mL/min	1.24	92.4	1.32	1210
Flow Rate: 1.9 mL/min	1.11	93.2	1.25	1188
Column Temperature: 28 °C	1.16	92.6	1.28	1325
Column Temperature: 32 °C	1.17	92.3	1.27	1346
Wavelength 222 nm	1.16	91.6	1.27	1322
Wavelength 226 nm	1.16	91.6	1.27	1322

**Table 11:** Solution stability for standard solution.

Time	Hydrocortisone Acetate Percent Agreement (%)	Hydrocortisone Acetate Percent Agreement (Standard)	Pramoxine HCl Percent Agreement (%)	Pramoxine HCl Percent Agreement (Standard)	Preservative Substance Percent Agreement (%)	Preservative Substance Percent Agreement (Standard)
**INITIAL**	100.0	100.0	100.0	100.0	100.0	100.0
**6th Hour**	100.3	100.3	100.3	100.1	100.3	100.1
**12th Hour**	101.2	101.1	100.9	100.8	101.2	101.2
**18th Hour**	102.2	100.9	101.9	100.5	102.6	101.0
**48th Hour**	108.3	104.8	106.9	105.8	113.8	103.6

**Table 12:** Compositions for the different formulations.

Ingredients	Ratio%						
	FT-1	FT-2	FT-3	FT-4	FT-5	FT-6	FT-7
Hydrocortisone acetate	2.5	2.5	2.5	2.5	2.5	2.5	2.5
Pramoxin HCl	1	1	1	1	1	1	1
Stearic acid	12	6	9	9	9	8	9
White vaseline	9	15	12	12	12	5	12
Isopropyl palmitate	2	2	5	2	2	4	1
Cetyl alcohol	~	~	~	~	~	~	~
Polyoxyl 40 stearate	~	~	~	~	~	~	~
Propylene glycol	~	~	~	~	~	~	~
Potassium sorbate	~	~	~	~	~	~	~
Sorbic acid	~	~	~	~	~	~	~
Liquid paraffin	~	~	~	~	~	~	~
Lanoline alcohol	1	1	0	0	1	1	0
Trietanolamin lauryl sulfate	1	1	1	1	1	1	1
Deionized water	q.s.	q.s.	q.s.	q.s.	q.s.	q.s.	q.s.
pH	2.5	5.2	4.0	4.0	4.0	4.5	4.0

FT: formulation trial. Some excipient amounts were kept constant in all formulas and indicated by the symbol ~.

**Table 13:** Qualitative and quantitative results of control tests.

Tests	Pramosone Cream 2.5%	Results						
		FT-1	FT-2	FT-3	FT-4	FT-5	FT-6	FT-7
**Appearance**	White, smooth semi-solid cream	*	*	White, smooth, semi-solid cream	White, smooth, semi-solid cream	White, smooth, semi-solid cream	White, smooth, semi-solid cream	White, smooth, semi-solid cream
**Content Uniformity**								
Hydrocortisone acetate	Max: 99.7% Min: 97.5%	Max: 129.6% Min: 88.3%	Max: 125.1% Min: 83.4%	Max: 109.3% Min: 102.1%	Max: 101.0% Min: 98.9%	Max:102.4% Min: 100.9%	Max: 99.8% Min: 98.6%	Max: 97.5% Min: 93.7%
Pramoxine HCl	Max: 98.9% Min: 98.0%	Max: 131.4% Min: 84.6%	Max: 126.0% Min: 82.8%	Max: 107.0% Min: 99.5%	Max: 100.2% Min: 99.7%	Max:99.6% Min: 98.3%	Max: 99.9% Min: 97.8%	Max: 95.0% Min: 92.1%
**pH**	4.0	2.5	5.2	4.0	4.0	4.0	4.5	4.0
**Viscosity (cp)**	270 × 10^3^	268 × 10^3^	268 × 10^3^	650 × 10^3^	268 × 10^3^	265 × 10^3^	272 × 10^3^	63 × 10^3^
**Assay**								
Hydrocortisone acetate	98.9%	118.9%	115.6%	108.7%	100.3%	101.2%	99.1%	95.3%
Pramoxine HCl	98.3%	120.9%	118.8%	105.3%	99.5%	100.4%	99.4%	93.9%
Antimicrobial preservative	99.7%	115.7%	113.1%	109.0%	103.3%	101.5%	100.7%	98.5%
**Related Compounds**								
Max. degradation product	0.15%	0.19%	0.18%	0.16%	0.15%	0.16%	0.16%	0.16%
Total impurity	0.19%	0.20%	0.21%	0.19%	0.18%	0.20%	0.19%	0.19%

* It was observed that the oil and water content in the cream separated and formed a two phase.

## Data Availability

The original contributions presented in the study are included in the article, further inquiries can be directed at the corresponding author.
